# Divergent hypersensitivity responses following topical application of the quaternary ammonium compound, didecyldimethylammonium bromide

**DOI:** 10.1080/1547691X.2017.1397826

**Published:** 2017-12

**Authors:** Hillary L. Shane, Ewa Lukomska, Aleksandr B. Stefaniak, Stacey E. Anderson

**Affiliations:** a Health Effects Laboratory Division, National Institute for Occupational Safety and Health, Morgantown, WV, USA; b Respiratory Health Division, National Institute for Occupational Safety and Health, Morgantown, WV, USA

**Keywords:** DDAB, hypersensitivity, allergic disease, quaternary ammonium compounds

## Abstract

Didecyldimethylammonium bromide (DDAB) is a fourth generation dialkyl-quaternary ammonium compound (QAC) that is used in numerous products for its antimicrobial properties. While many QACs have been associated with allergic disease, the toxicity and sensitization of DDAB have not been thoroughly investigated. The purpose of these studies was to evaluate the irritancy and sensitization potential of DDAB following dermal application in a murine model. DDAB induced significant irritancy (0.0625–2%), evaluated by ear swelling in female BALB/c mice. Initial evaluation of the sensitization potential was conducted using the local lymph node assay (LLNA) at concentrations ranging from 0.0625% to 2%. A concentration-dependent increase in lymphocyte proliferation was observed with a calculated EC3 value of 0.057%. Immune cell phenotyping along with local and systemic IgE levels were evaluated following 4 and 14 days of dermal application. Phenotypic analyses revealed significant and dose-responsive increases in the absolute number of B-cells, CD4^+^ T-cells, CD8^+^ T-cells, and dendritic cells in the draining lymph nodes (DLNs) following 4 and 14 days of dermal exposure with significant increases in the number of activated B-cells and dendritic cells. However, increased activation of CD4^+^ T-cell and CD8^+^ T-cells was only observed following four days of DDAB exposure. Exposure to DDAB also induced increased production of IgE as evaluated by phenotypic analysis of DLN B-cells (IgE^+^ B-cells) and measurement of total serum IgE levels following 14 days but not four days of dermal application. Significant increases in gene expression were observed in the DLN (*Il-4*, *Il-10*, and *ox40l*) and ear (*tslp*) following 4 and 14 days of DDAB exposure. These results demonstrate the potential for development of irritation and hypersensitivity responses to DDAB following dermal exposure and raise concerns about the effects of exposure duration on hypersensitivity responses.

## Introduction

Quaternary ammonium compounds (QAC) have been used for over 50 years as water-based surface disinfectants due to their low volatility and broad antimicrobial capabilities (Zhang et al. 2015). There are many different QAC that are increasingly being used in hospitals, hotels, and in consumer products. They are primarily used as cleaners and disinfectants for non-critical surfaces, algaecides, fabric softeners, antistatic agents, and wood preservatives (Rajkowska et al. 2016). However, in hospitals and other health care settings, they are frequently used as high-level disinfectants for the decontamination of surfaces, surgical instruments, endoscopes, and other medical instruments (Saito et al. 2015). In addition, the use of QAC as antiseptics, disinfectants, detergents, and preservatives has increased their incorporation into consumer products that are applied to the skin or eyes for the purpose of decreasing microbial contamination and reducing the incidence of pathogen-induced illness.

All QAC are permanently charged ions with four alkyl side chains. Their structure contains at least one hydrophobic hydrocarbon chain linked to a positively charged nitrogen atom, and other alkyl groups which are mostly short-chain substitutes. The biocidal activity is conferred through alkyl chain length (McBain et al. 2004) and the chemistry and formulation of QAC are constantly changing to promote or boost activity against intrinsically resistant groups of microorganisms (Carson et al. 2008). Several generations of QAC have evolved since they were first introduced (Gerba 2015). These generations are defined by substitutions of the side chains designed to increase biocidal activity and sometimes are the result of combining other generations (dual QAC). More recently developed, the fourth generation of QAC is twin chain compounds or dialkyl QAC that allow for a wide spectrum of activity. These new synthetic polymeric QAC contain multiple positively charged amine centers that confer antimicrobial, anti-static, and surfactant properties in solution. They have stronger antimicrobial ability compared to their single chain predecessors which include improved tolerance to anionic surfactants, protein soil and water hardness salts. In an effort to reduce the incidence of pathogen induced illnesses, newer generations of QAC are continually being developed.

Throughout their 50+ years of use, QAC have generally been regarded as safe; however, there is very limited published research describing the toxicity of these compounds, especially regarding the newer generations. Of concern, several recent studies have shown that the use of QAC in animal facilities has led to decreased rates of fertility and result in birth defects in mice (Melin et al. 2014, 2016; Hrubec et al. 2017), highlighting the need for further research on their potential effects on health. QAC have been identified to be among the most common allergens in the health care profession and have been associated with both contact dermatitis and work related asthma (Bernstein et al. 1994; Shaffer and Belsito 2000; Suneja and Belsito 2008). While there have been fewer overall reported cases of sensitization to the newer formulations of QAC, delayed and immediate-type allergic reactions have been recently reported (Dejobert et al. 1997; Dibo and Brasch 2001; Houtappel et al. 2008; Ruiz Oropeza et al. 2011; Mowitz and Ponten 2015).

While a role for QAC in allergic disease has been suggested, the exact mechanism of sensitization to these compounds remains to be investigated. Previously, the irritancy and sensitization potential of didecyl-dimethylammonium chloride (DDAC), one of the newer fourth generation QAC that has been described as a sensitizer in epidemiology studies, were evaluated (Anderson et al. 2016). DDAC induced significant irritancy (0.5 and 1%), evaluated by ear swelling in female BALB/c mice and was identified as a sensitizer with a calculated EC3 value of 0.17% (local lymph node assay (LLNA)). Dermal application (four days) of DDAC did not induce increased production of local or total IgE. In addition, there was also a significant and dose-responsive increase in the number of activated CD4^+^ T-cells, CD8^+^ T-cells, B-cells, and dendritic cells, suggesting a T-cell-mediated hypersensitivity response.

Didecyldimethylammonium bromide (DDAB) is another fourth generation QAC that is increasingly being used as a broad-spectrum bactericide and fungicide. It causes disruption of intermolecular interactions and dissociation of lipid bilayers, making it extremely effective against bacteria, algae, and molds (Rajkowska et al. 2016). It is recommended for use in the food and agriculture industries and is also used as a disinfectant cleaner for linen in hospitals and hotels industries. Due to the high potential for human exposure, epidemiological studies suggesting an association with allergic disease, and the lack of dermal toxicological data, this study was performed to evaluate the irritancy and skin sensitization potential of DDAB using a murine model in an effort to assess its role in the development of allergic disease.

## Materials and methods

### Animals

Female BALB/c mice were used for the murine models as this strain has a T-helper (T_H_)-2 bias, is comparable to the CBA mouse in responses in the LLNA (Woolhiser et al. 2000), and is commonly used to evaluate IgE-mediated sensitization (Klink and Meade 2003). Mice were purchased from Taconic (Germantown, NY) at 6–8 weeks of age and upon arrival, allowed to acclimate for a minimum of five days. Each shipment of mice was randomly assigned to treatment group, weighed, and individually identified via tail marking using a permanent marker or tattoo. A preliminary analysis of variance (ANOVA) on body weights was performed to ensure homogeneous distribution across treatment groups. Mice were housed (at a maximum of five per cage) in ventilated plastic shoebox cages with hardwood chip bedding, and both NIH-31 modified 6% irradiated rodent diet (Harlan Teklad, Madison, WI) and tap water from water bottles were available *ad libitum*. Temperature in the animal facility was maintained at 68–72 °F and relative humidity at 36–57%. A light/dark cycle was maintained on 12-h intervals. All animal experiments were performed in the AAALAC International accredited NIOSH animal facility in accordance with an animal protocol approved by the Institutional Animal Care and Use Committee.

### Test articles

**Figure 1.**
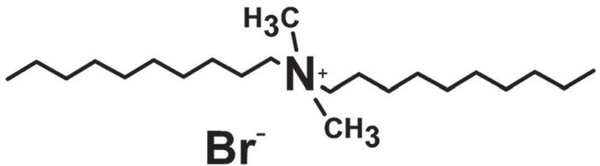
Chemical structure of DDAB.

Didecyldimethylammonium bromide (CAS# 2390–68-3) (Figure 1), acetone, and α-hexylcinnamaldehyde (HCA; CAS# 101–86-0) were purchased from Sigma (Milwaukee, WI).

### Concentration range finding studies

Concentration range finding studies were performed to select levels of DDAB to be used for dermal exposures. Mice (three/ group) were exposed topically to acetone vehicle or increasing concentrations of DDAB (up to 10%) in acetone on the dorsal surface of each ear (25 μl/ear) for three consecutive days. Acetone was selected as the appropriate vehicle based on solubility, historical control data, and accepted use in skin sensitization studies (NIEHS 1999). Animals were allowed to rest for two days following the final exposure and then weighed and examined for signs of overt toxicity including loss of body weight, fatigue/lack of activity, and ungroomed fur.

### Combined irritancy and local lymph node assay

To determine DDAB irritancy and sensitization potential, a combined LLNA was conducted as described in Anderson et al. (2007) and according to the method in the ICCVAM Peer Review Panel report (NIEHS 1999), with minor modifications. In brief, mice (five/group) were exposed topically to acetone vehicle, increasing doses of DDAB (0.0625–2.0%), or positive control (30% HCA) on the dorsal surface of each ear (25 μl/ear) for three consecutive days. This level of HCA is an accepted well-characterized positive control for the LLNA (NIEHS 1999). For irritancy evaluation, thickness of the right and left ear pinnae of each mouse was measured using a modified engineer’s micrometer (Mitutoyo Co., Aurora, IL) before the first chemical administration and 24 h post-final exposure. Mean percentage ear swelling was calculated as: 100 × [(mean post-challenge ear thickness ‒ mean pre-challenge ear thickness)/mean pre-challenge thickness]. Animals were allowed to rest for two days following the final exposure and then (Day 6) injected intravenously via the lateral tail vein with 20 μCi [^3^H]-thymidine (specific activity 2 Ci/mmol; PerkinElmer, Shelton, CT). These mice were euthanized 5 h later via CO_2_ asphyxiation, and the left and right auricular draining lymph nodes (DLNs; drain site of chemical application) located at the bifurcation of the jugular vein were excised and pooled for each animal. Single cell suspensions were made, incubated overnight in 5% trichloroacetic acid, and samples then counted in a Tri-Carb 2500TR liquid scintillation analyzer (Packard, Franklin Lakes, NJ). Stimulation indices (SIs) were calculated by dividing the mean disintegrations per minute (DPM) per test group by the mean DPM for the vehicle control group. EC3 values (concentration of chemical required to induce three-fold increase over vehicle control) were calculated based on Basketter et al. (1999). Dosing levels (0.0625–2.0%) were selected based on the results from these studies.

### Phenotypic analysis of draining lymph node cells

For phenotypic analysis, mice (five per group) were exposed to 25 μl/ear of acetone or increasing concentrations of test article (0.0625%, 0.125%, 0.25% DDAB), once daily for 4 (four days) or 14 consecutive days (14 days). Mice were euthanized (by CO_2_) on Day 10 (four days) or Day 15 (14 days), weighed, and examined for gross pathology. The liver, spleen, kidneys, and thymus were removed, cleaned of connective tissue and weighed (14 days only). Serum was collected for total IgE analysis (see below). DLN cell suspensions (two nodes/animal/3 ml PBS [phosphate-buffered saline, pH 7.4]) were prepared by mechanical disruption of tissues between frosted microscope slides. Live cells were counted on a Cellometer (Nexcelom Bioscience, Lawrence, MA) using acridine orange/propidium iodide staining solution. Cells (1–2 × 10^6^) were then placed in 96-well U-bottom plates, washed in staining buffer (PBS +1% bovine serum albumin (BSA) + 0.1% sodium azide [both Sigma, St. Louis, MO]), and then suspended in staining buffer containing anti-mouse CD16/32 antibody (clone 93) to block surface F_c_ receptors (eBioscience, San Diego, CA).

Cells were then incubated for 30 min at 4 °C in a cocktail of fluorochrome-conjugated antibodies against specific surface antigens, including IgE-FITC (R35–72), B220-V500 (RA3–6B2), CD8a PE (53–6.7) (BD Biosciences, San Jose, CA), CD4-BV-605 (GK1.4) (BioLegend, San Diego, CA), CD11b-PerCP-Cyanine5.5 (M1/70), CD11c-eF-450 (N418), CD25 PE-eF-601 (PC61.5), CD86-APC (GL1), MHC II-AF700 (M5/14.15.2), and CD44-eFluor 780 (IM7) (eBioscience, San Diego, CA). Cells were then washed in staining buffer, fixed in Cytofix buffer (BD Biosciences, San Jose, CA) and within 24 h, suspended in staining buffer for analysis in a BD Biosciences LSR II flow cytometer (San Jose, CA), collecting 100,000 events/sample. Compensation controls were prepared with eBioscience (San Diego, CA) UltraComp eBeads. The IgE^+^B220^+^ (IgE^+^ B-cells) populations were analyzed as described by Manetz and Meade (1999). Data analysis was performed using FlowJo v10 software (TreeStar Inc., Ashland, OR), and the gating strategy shown in Supplemental Figure 1.

### Serum IGE antibody levels

From mice included in the phenotyping study, blood samples were collected via cardiac puncture. Sera were separated by centrifugation and frozen at –20 °C for subsequent analysis of total IgE. A standard colorimetric sandwich ELISA was performed according to manufacturer directions (Mouse IgE Ready-Set-Go!, eBioscience, San Diego, CA). The lower limit of sensitivity was 4 ng/ml.

### Ear and draining lymph node gene expression

Total RNA was isolated from the DLN (miRNeasy kit) and ear (mRNeasy kit) according to manufacturer directions (Qiagen, Germantown, MD). A QiaCube (Qiagen, Germantown, MD) automated RNA isolation machine was utilized in conjunction with the specified RNA isolation kit. The concentration/purity of the isolated RNA was determined using an ND-1000 spectrophotometer (Thermo Scientific Nanodrop, Wilmington, DE). For gene analysis, first strand cDNA synthesis was performed using a High-Capacity cDNA Synthesis Kit (Applied Biosystems, Carlsbad, CA) according to manufacturer recommendations.

For mRNA analysis, TaqMan Universal Fast master mix (Life Technologies, Carlsbad, CA), cDNA, and mouse-specific mRNA primers (TaqMan Custom PCR Arrays) were combined and PCR was performed according to the manufacturer protocols (TaqMan Gene Expression Analysis). Primers used include: *β-actin*, *runx1*, *tbet*, *gata3*, *foxp3*, *Il-4*, *Il-5*, *Il-10*, *Il-13*, *ox40l*, *tslp*, and *Il-6*. MicroAmp Fast Optical 96-well reaction plates were analyzed in a 7500 Fast Real Time PCR system (Applied Biosystems, Carlsbad, CA) using cycling conditions specified by the manufacturer. β-Actin was used as the endogenous reference control gene as expression was determined to be stable following chemical exposure (data not shown). RT-PCR data were collected and represented as relative fold-change over control calculated from: 2^‒ΔΔCt^=ΔCt_Sample_‒ΔCt_Control_. ΔCt = Ct_Target_‒Ct_β-ACTIN_, where Ct = cycle threshold (defined by manufacturer).

### Statistical analysis

For analysis, data were first tested for homogeneity using Bartlett’s Chi Square test. If homogeneous, a one-way ANOVA was conducted. If the ANOVA showed significance at *p* ≤ 0.05, Dunnett’s multiple range *t*-test was used to compare treatment groups with the control group. Linear trend analysis was performed to determine if DDAB had exposure concentration-related effects for the specified endpoints. Statistical analysis was performed using Prism v.5.0 (Graph Pad, San Diego, CA). Statistical significance is designated by *p ≤ 0.05 and **p ≤ 0.01.

## Results

### In vivo *studies identify DDAB to be immune sensitizer and irritant*

**Figure 2.**
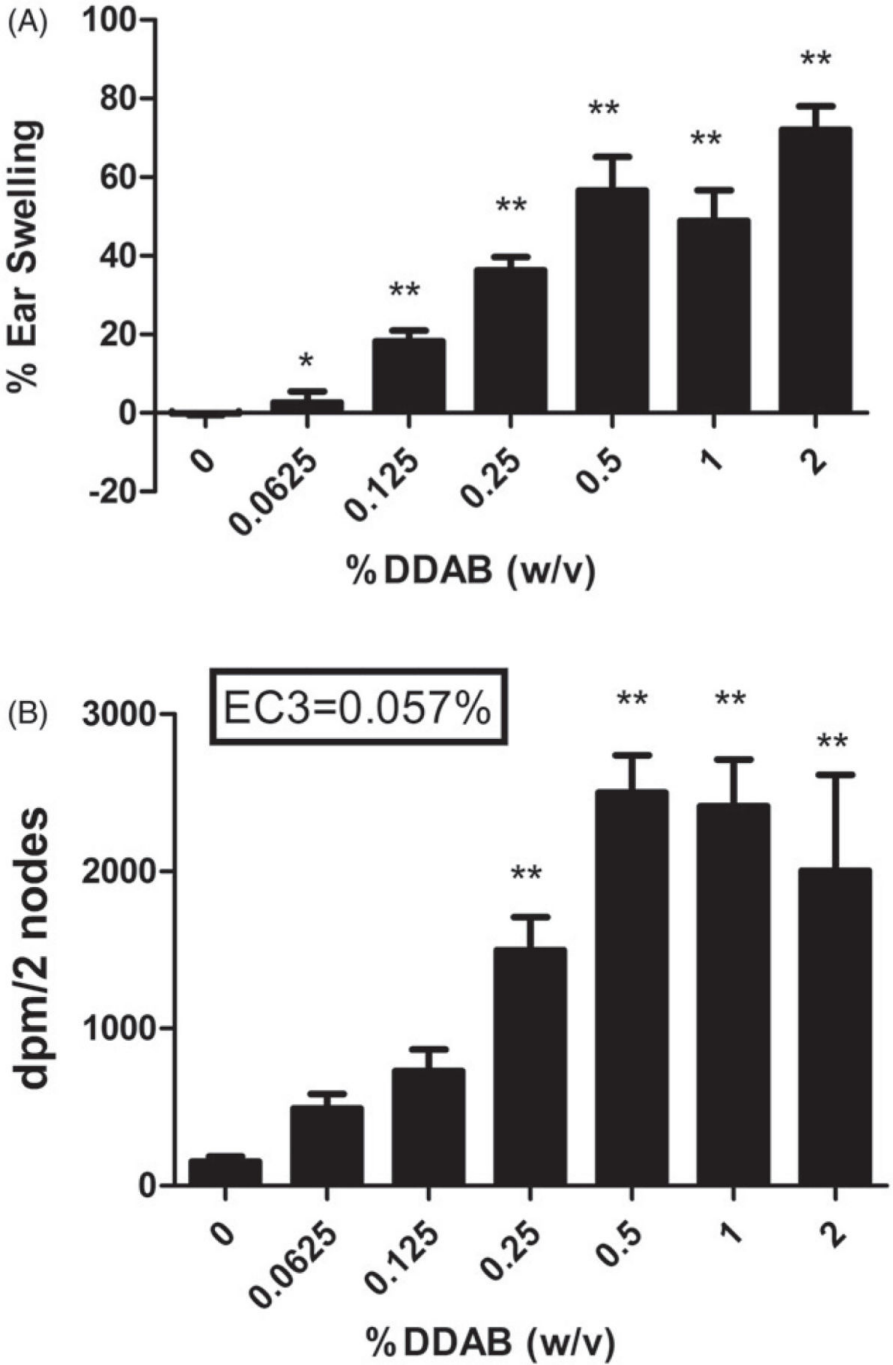
Irritancy and allergic sensitization potential after dermal exposure to DDAB. Analysis of irritancy (A) and allergic sensitization potential (B) of DDAB using the LLNA. Irritancy was determined using measurements collected at 24 hours following the final DDAC exposure (three days). DPM represents [^3^H]-thymidine incorporation into DLN cells of BALB/c mice following exposure to vehicle or concentration of DDAC (0.0625–2%). SI value is the stimulation index (fold change over vehicle control). Bars represent mean (±SE) of five mice per group. Significantly different from acetone controls at *p < 0.05 or **p < 0.01.

In the initial range finding studies, exposure to 2% DDAB for three days resulted in less than 10% loss in body weight and did not result in signs of overt toxicity or visual signs of excessive inflammation at the exposure sites (data not shown). Therefore, this concentration was selected as the highest concentration used in the LLNA and irritancy studies. Topical application of all concentrations of DDAB induced significant ear swelling 24 h post final chemical exposure (three days) ranging from ≈5% (0.0625% DDAB) to 70% (2% DDAB) (Figure 2(A)). DDAB also tested positive in the LLNA and an EC3 value of 0.057% was calculated as previously described (Figure 2(B)). A dose responsive increase (Linear Trend Test *p* < 0.05) in lymphocyte proliferation was observed following exposure to DDAB reaching statistical significance at 0.25%. HCA (30%) was used as a positive control for these experiments and resulted in a SI value of 12 (data not shown).

### Repeat DDAB application results in reduced thymic weights

To avoid potential overt toxicity, reduced concentration (0.0625–0.125%) was used in repeat exposure studies to evaluate DDAB (Table 1). Following 14 days of dermal exposure, no significant decreases in body weight were observed. However, a statistically significant decrease in both thymus weight and thymus as a percent of body weight was observed following exposure to 0.25% DDAB (Table 1). These decreases were dose-responsive (Linear Trend Test *p* < 0.05). No other changes in organ weights were observed.

### Exposure to DDAB induced increased DLN cellularity consisting of activated leukocytes

Dermal treatment with DDAB resulted in dose-dependent increases in DLN cellularity statistically significant at 0.125% and 0.25% following four days of exposure (Table 2), and at all concentrations following 14 days of exposure (Table 3). There was almost a five-fold increase in cellularity following exposure to 0.25% DDAB (1.29 × 10^7^) compared to vehicle (0.28 × 10^7^) for the four-day application and almost a six-fold increase in cellularity following exposure to 0.25% DDAB (1.97 × 10^7^) compared to the vehicle (0.348 × 10^7^) for the 14-day exposure. Exposure to 0.125% and 0.25% DDAB produced statistically significant increases in the absolute number of B-cells, CD4^+^ T-cells, CD8^+^ T-cells, and dendritic cells for the four-day application (Table 2). For the 14-day application, statistically significant increases were observed for absolute numbers of B-cells (0.125% and 0.25%), CD4^+^ T-cells (0.625%, 0.125%, 0.25%), CD8^+^ T-cells (0.625%, 0.125%, 0.25%), and dendritic cells (0.125% and 0.25%) (Table 3). Statistically significant increases in the percent of the B-cell population were also observed (0.125% and 0.25% DDAB) following 4 and 14 days of application as previously described. In addition, significant decreases in the percent of CD4^+^ and CD8^+^ T-cells were observed following treatment with DDAB for 4 days and 14 days. Interestingly, the changes in CD4 T-cell frequency were only observed at 0.25% for the 14-day study compared to 0.125 and 0.25% for the four-day study.

**Figure 3.**
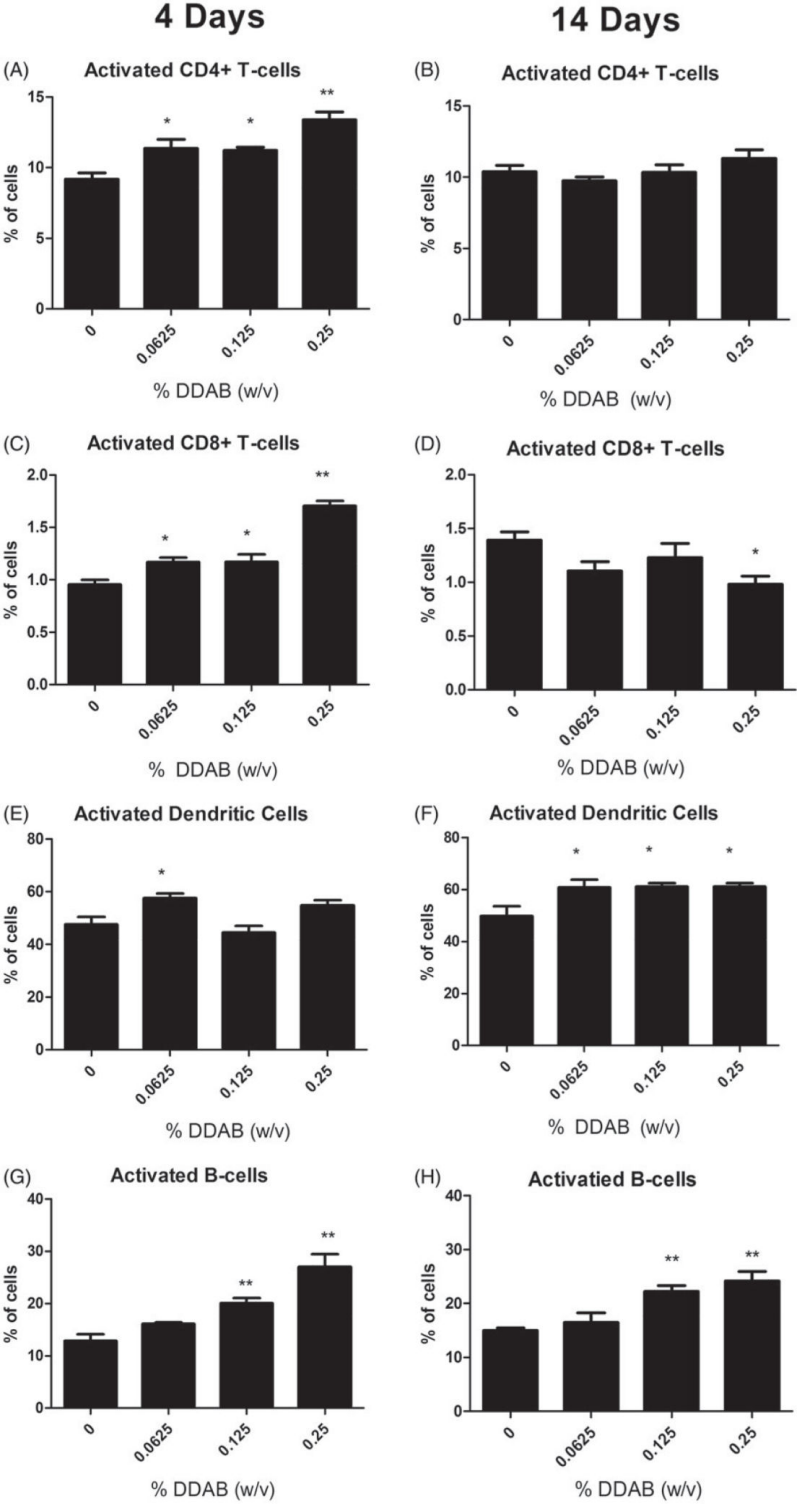
Increases in activated leukocytes following dermal exposure to DDAB. Analysis of percent CD44-high CD4^+^ (A), CD44-high CD8^+^ (C), CD86^+^ dendritic cells (E), and CD86^+^ B-cells (G) of total lymphocytes on Day 10 following four days of DDAC application. Analysis of percent CD44-high CD4^+^ (B), CD44-high CD8^+^ (D), CD86^+^ dendritic cells (F), and CD86^+^ B-cells (H) of total lymphocytes on Day 15 following 14 days of DDAC application. Bars represent mean (±SE) of five mice per group. Significantly different from acetone controls at *p < 0.05 or **p < 0.01.

Exposure to DDAB did not alter the percentage of dendritic cells identified by a high CD11b and MHC II surface marker expression for either exposure duration. Activated cell percentages were also evaluated based on co-expression of surface markers CD44 (CD4^+^ and CD8^+^ T-cells) or CD86 (dendritic and B-cells). Significant increases in the percent of activated CD4^+^ (0.0625%, 0.125%, 0.25%), CD8^+^ (0.0625%, 0.125%, 0.25%), B-cells (0.125%, 0.25%), and dendritic cells (0.0625%) were noted following the four-day treatment with DDAB (Figure 3(A,C,E,G)). Interestingly, 14 days of DDAC treatment did not elevate the percent of activated CD4^+^ cells and the CD8^+^ population was significantly reduced at the 0.25% level (Figure 3(B,D)). In addition, significant increases in percentages of activated dendritic cells were observed at all concentrations (Figure 3(F)); the percent of activated B-cells was similar to what was observed following four days of DDAB application (Figure 3(H)).

### Repeat exposure to DDAB induced an increase in local and systemic IgE levels

To further evaluate the mechanisms of the hypersensitivity response, the percent of B-cells and IgE^+^B220^+^ cells (IgE^+^ B-cells) in the DLN was determined using flow cytometry. Soluble IgE bound to the B-cell surface via the low affinity IgE receptor (CD23) is dependent on the level of soluble IgE present in the local DLN environment (representative of local IgE levels) and changes in this population following allergen exposure have been detected earlier than serum IgE levels. Manetz and Meade (1999) have shown that select chemicals capable of inducing T_H_2-mediated allergic responses, have similar peak increases in the percent IgE^+^ B-cells and total B-cell (B220^+^) populations and become significantly elevated at equivalent concentrations of the test chemical. Consistent with the LLNA results, statistically significant and dose-responsive (Linear Trend Test *p* < 0.01) increases in the numbers and percent of B-cells were observed following exposure to 0.125% and 0.25% DDAB for both exposure durations (Table 4). Statistically significant increases in the percent and number of IgE^+^ B-cells were also observed in the DLN of mice treated with 0.25% DDAB for four days.

**Figure 4.**
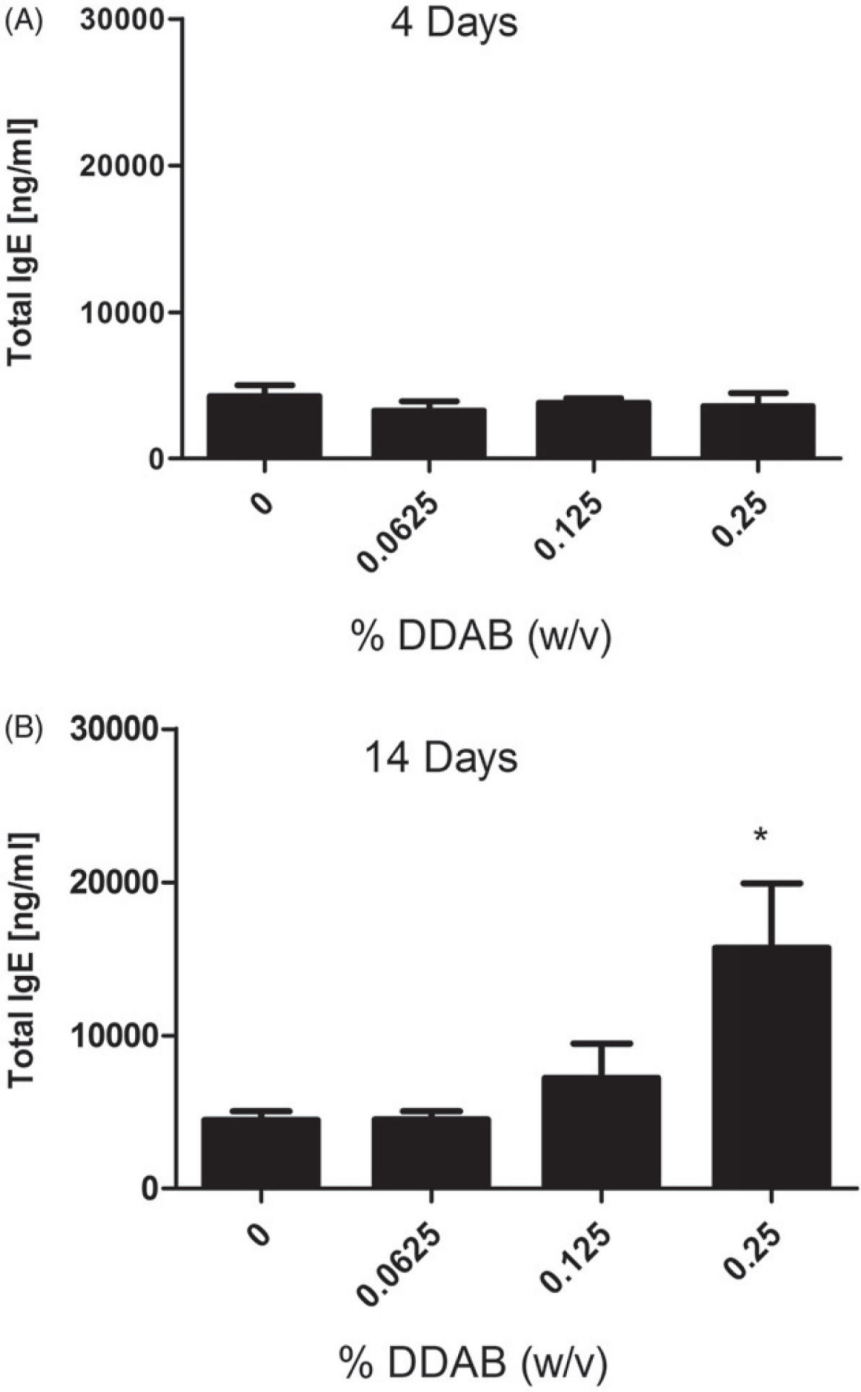
Systemic IgE levels following dermal DDAB exposure. Analysis of total serum IgE on (A) Day 10 following four days of DDAB application and (B) Day 15 after 14 days of DDAB application. Bars represent mean (±SE) of five mice per group. Significantly different from acetone controls at *p < 0.05.

In addition, there was a significant increase in percent (0.25%) and number of IgE^+^ B-cells (0.125% and 0.25%) for the mice treated with DDAB for 14 days (Table 4). The percent IgE^+^ B-cell population (~30%) and total B-cell population (~34%) were similar following the 14-day 0.25% DDAB exposure but not for the four-day exposure (19% IgE^+^ B-cells vs. 33% B-cells) suggesting a potential T_H_2 mediated response for the longer exposure duration. Serum IgE is commonly used as an indicator of T_H_2 hypersensitivity responses. Supporting the phenotyping results, exposure to DDAB for 14 days, but not four days, produced a significant elevation in total serum IgE levels compared to the vehicle control group (Figure 4). Exposure to 0.25% DDAB resulted in a significant elevation of total serum IgE (15 702 ng/ml) compared to the vehicle control (4479 ng/ml).

### Repeat application with DDAB induced increases in allergic cytokine gene expression in the draining lymph node and ears

**Figure 5.**
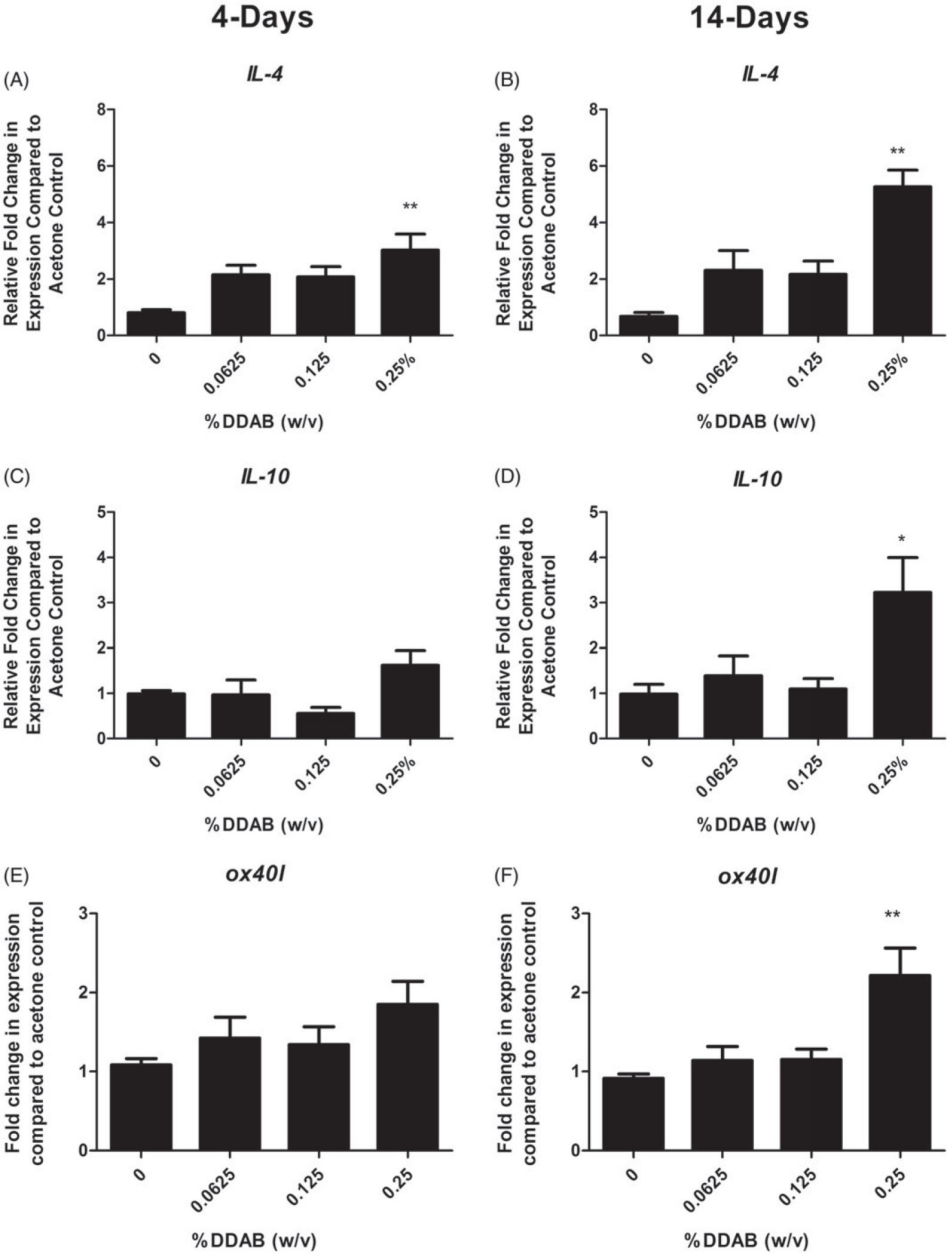
Increases in lymph node gene expression following DDAB application. Gene expression of *IL-4*, *IL-10*, and *ox40l* in lymph nodes following DDAB application for four days (A, C, E) or 14 days (B, D, F). Bars represent mean (±SE) of five mice per group. Significantly different from acetone controls at *p < 0.05 or **p < 0.01.

**Figure 6.**
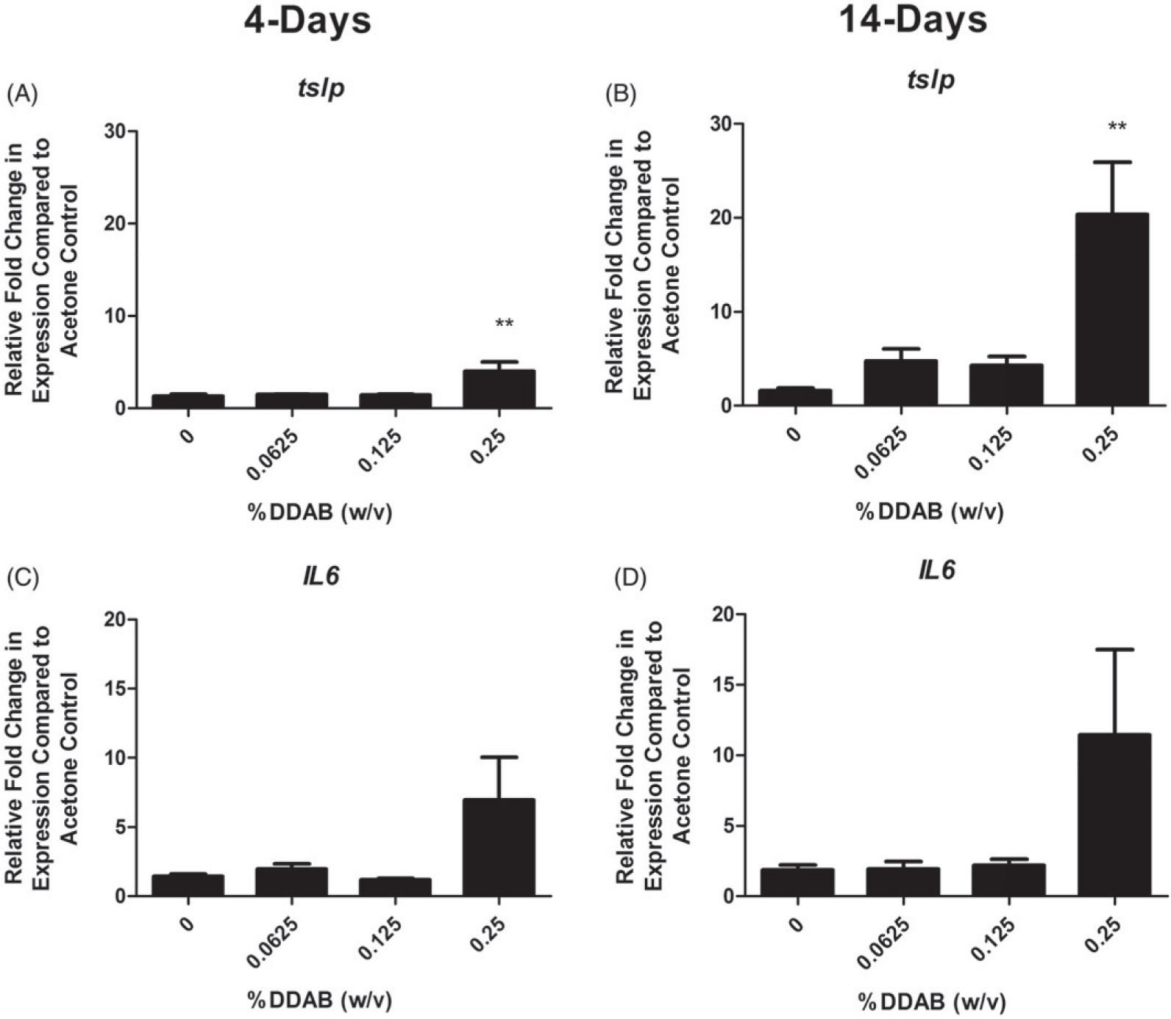
Increases in ear gene expression following DDAB application. Gene expression of *tslp* and *IL-6* in the ear following DDAB application for four days (A, C) or 14 days (B, D). Bars represent mean (±SE) of five mice per group. Significantly different from acetone controls at **p < 0.01.

LN and ears were evaluated for expression of cytokines and signaling molecule that are capable of influencing allergic disease in an attempt to better understand the mechanism of action. In the LN, supporting a T_H_2-mediated allergic mechanism, a significant fold increase in *Il*-4 compared to the vehicle control was observed following both 4 (~3) and 14 (~5) days of 0.125% DDAB treatment (Figure 5(A,B)). *Il-10* and *ox40l* were both significantly elevated but only for the 14-day 0.25% DDAB treatment (Figure 5(C–F)). The ears were also evaluated for changes in gene expression (Figure 6). Significant fold-increases in *tslp* were observed following both four days at 14 days of DDAB exposure (Figure 6(A,B)), while no significant changes were observed in levels of the T_H_2 cytokines at this site. While these changes were only significant for the 0.25% concentration, elevated (but not significant) fold-changes were noted at the lower concentrations for the 14-day study. In addition, the overall increase in fold-change was much greater for the longer exposure (4 vs. 20 for *tslp*). While there was increased *ll-6* expression at the high concentration, significance was not reached (Figure 6(C,D)). No significant changes in *runx1*, *tbet*, *gata3*, *foxp3*, *Il-5*, *Il-10*, or *Il-13* were seen (data not shown).

## Discussion

Millions of people suffer from allergic conditions characterized by exaggerated immune responses. Contact dermatitis is the second most commonly reported occupational illness accounting for 10–15% of all occupational diseases and urticaria (Anderson and Meade 2014). Health care is one of the occupational sectors with the highest incidence of allergic disease (Warshaw et al. 2008) and exposure to cleaning agents has been identified as one of the most common causes of allergic disease in this sector (Pechter et al. 2005). Cleaning products that contain QAC and other disinfectants are commonly used to prevent the spread of serious infectious disease and their use is encouraged in clinical and other health care settings to prevent the transmission of pathogens. However, in recent years, there has been a rise in publications linking asthma to the use of cleaning products.

Skin is thought to be a significant route of exposure for DDAB and other QAC (Buist et al. 2007). Working concentrations of QAC in disinfectants and cleaning products typically range from 0.01% to 1%, but can be as high as 5% which is a similar range to the concentrations tested in the present study (Bank, Hazardous Substances Data 2010). In addition, the concentrated solutions used for dilution can contain ≥25% of specific QAC and there is the potential for skin exposure to high concentrations as a result of splashes and spills. While manufactures typically regard these products as safe and non-sensitizing, specific toxicological information is often lacking in the SDS since it is not required for QAC or other chemicals to be listed if they are ≤1% of the cleaning product (Saito et al. 2015). However, DDAB was demonstrated in the present study to be a strong sensitizer/irritant at concentrations much less than 1% raising concerns about potential exposure to this and other QAC.

The studies described here use a standardized murine model to begin to evaluate the sensitization potential of a QAC commonly used in cleaning products, DDAB. DDAB was identified as an irritant and strong sensitizing chemical. In addition, 14 days of exposure to 0.25% DDAB resulted in a significant reduction in thymus weights, while no other overt signs of toxicity were observed. As thymus weight loss is one of the most sensitive indicators of immunosuppression caused by toxicants (Pearse 2006), it is possible that continued exposure to DDAB (beyond 14 days) may result in suppression of immune responses and more overt signs of toxicity. However, in these studies – where body weights and other organ weights were unchanged – it is unlikely that reduction in thymus weights observed had biological significance with respect to sensitization within the studied timeframe. In an effort to examine the mechanism of sensitization and potential toxicity of DDAB, two exposure durations (four days and 14 days) were examined. Typically, chemical sensitizers are low molecule weight (LMW) molecules that are categorized as dermal or respiratory sensitizers, depending on the route of sensitization. Dermal sensitizers tend to result in allergic contact dermatitis (ACD) while respiratory sensitizers induce asthma. Although dermal and respiratory sensitization typically predicts the type of allergic response induced (T-cell vs. IgE, respectively), this paradigm is not always valid. However, for the purpose of this manuscript, IgE and T-cell-mediated hypersensitivity mechanisms will be identified as T_H_2 and T_H_1 responses, respectively.

This laboratory has published data on numerous chemicals, generally classified as T_H_2-mediated or IgE-mediated chemical sensitizers, including *o*-phthalaldehyde (OPA), glutaraldehyde, and toluene diisocyanate (TDI), that have induced elevations in IgE (local and total) at 10 days following four days of chemical treatment (Azadi et al. 2004; Anderson et al. 2010, 2016). In the present study, antibody levels were also examined following 14 days of continuous chemical application. Interestingly, based on the paradigm described by Manetz and Meade (1999) and total IgE levels, the results suggest a T_H_2-mediated mechanism for the 14 days but not the four-day exposure duration. While the LLNA EC3 value has been established as a threshold value for hazard identification for sensitization, several studies have shown that exposure duration and concentration may also play a role. For example, extended exposure to doses below the EC3 value have been shown to induce SI values above 3 for select formaldehyde donors (de Jong et al. 2007), suggesting that duration of exposure and a possibility of local chemical accumulation should be considered.

In addition to the IgE, significant differences were observed in the immunophenotyping profile for the two exposure durations. In general, exposure to DDAB produced statistically significant increases in absolute numbers of B-cells, CD4^+^ T-cells, CD8^+^ T-cells, and dendritic cells for both exposure durations. Furthermore, statistically significant decreases in the percent CD8^+^ T-cell populations were observed following exposure to 0.125% and 0.25% DDAB for four days; however, there was a slight but significant increase in this population at 0.25% DDAB for the 14-day exposure. Interestingly, while statistically significant increases in the percent of activated CD4^+^, CD8^+^, B-cells, and dendritic cells were observed following the four-day exposure to DDAB, 14 days of DDAB exposure did not elevate the percent of activated CD4^+^ cells and the CD8^+^ population was significantly reduced at the 0.25% concentration. It should be noted that due to migratory properties of T-cells, the differences in observed frequencies of activated cells may be due to egress of activated T-cells from the DLN to the site of chemical application (i.e., the ear).

Evaluation of DDAC showed similar trends for sensitization potential, IgE and immunophenotyping (surface markers and activation status) as DDAB; however, only the four-day exposure period was examined in the study and higher concentrations of DDAC (0.25–1%) were used compared to DDAB (Anderson et al. 2016). These findings suggest that the hypersensitivity responses induced by DDAB might have different mechanisms and mediators based on exposure duration; while not analyzed in this study, it is possible that DDAC and other QAC may have similar exposure/duration mediated divergent mechanisms.

To further explore hypersensitivity responses induced by DDAB, gene expression was also examined in the DLN and ear following both application timepoints. IL-4 is crucial for IgE expression because it is required for increased expression of CD23 on the B-cells, B-cell proliferation, isotype switching, and IgE synthesis, and its expression often supports polarization to a T_H_2 hypersensitivity response. While significant increases in *Il-4* were observed at both time points, the fold-increase was greater for the longer exposure duration supporting a more pronounced T_H_2 response. Increases in *Il-10*, another T_H_2 cytokine that has been shown to have a role in allergic disease (Zuska-Prot et al. 2016), were also observed following the extended DDAB application at high concentration. In addition, secretion of IL-10 mediates the function of T_reg_ cells (Sojka et al. 2008; Corthay 2009; Kimber et al. 2012) which have demonstrated a critical role in the development of immune tolerance by serving as effectors helping to prevent overzealous allergic responses (Sojka et al. 2008; Yadav et al. 2012). The observed augmentation in *Il-10* support a potential role for T_reg_ cells in the divergent hypersensitivity responses resulting from DDAB exposure.

While development of immune responses is often thought to be orchestrated in the DLN, and is thus largely studied at these sites, there is increasing data suggesting that early events occurring at the site of antigen contact may also be important. In addition to protection from the outside environment, as the largest organ in the body, the skin is progressively being recognized as an important player in relation to allergic disease (Deckers et al. 2017). This laboratory has previously investigated factors in the skin that may influence the immunological microenvironment and found that increased TSLP expression in the skin promotes T_H_2 allergic responses following dermal exposure to triclosan anti-microbial (Marshall et al. 2015). TSLP is expressed by different cell types present in the skin including epithelial cells, keratinocytes and dendritic cells (Ziegler et al. 2013) and is induced as an integral part of cellular and immune responses to skin barrier damage (Angelova-Fischer et al. 2010; Oyoshi et al. 2010). In the present study, *tslp* expression was significantly elevated following exposure to the high concentration of DDAB for both 4-day and 14-day application study. However, much higher levels (~4-fold vs. ~20-fold) were observed for the longer exposure duration, while *lL-6*, indicative of overall inflammation was relatively similar between the two exposure durations. It has been reported that TSLP activates dendritic cells to prime T_H_2 cells (Soumelis et al. 2002) in part through OX40-OX40L interactions (Ito et al. 2005). OX40L is an important costimulatory molecule for promoting effector and memory T-cell responses in allergic disease (Jember et al. 2001; Salek-Ardakani et al. 2003; Seshasayee et al. 2007). *Ox40l* was also significantly elevated in the DLN but only at the high concentration of DDAB and for the longer exposure duration. However, while *Il-4* was elevated in both exposure scenarios, the high levels of *tslp* and *ox40l* observed in the long duration correlate with the IgE levels observed in the phenotyping studies, suggesting skewing toward a T_H_2-mediated response. In addition to exposure to DDAB resulting in sensitization to the chemical, the high levels of TSLP produced at the site of application may indicate that DDAB can act as an adjuvant to promote allergic sensitization. Previously, it was shown that triclosan, while not sensitizing itself, increased the allergic potential of other allergens and increased asthmatic symptoms in an OVA model of allergy (Anderson et al. 2013) through a TSLP-mediated pathway (Marshall et al. 2015). Therefore, increases in respiratory diseases correlated with QAC use may occur through direct or indirect pathways; the mechanisms behind these observations warrant further investigation.

Collectively, these findings support a potential for multiple hypersensitivity mechanisms in response to dermal DDAB application. While a lack of increase in both local and total IgE along with an increased percent of activated CD8^+^ T-cells in the DLN following four days of exposure suggests that DDAB may induce a T_H_1-mediated hypersensitivity response, a T_H_2 response is suggested for the longer exposure duration. Although, T_H_2 responses are supported for both exposure durations when examined on a transcript level. Admittedly, the understanding of immunologic mechanisms underlying many hypersensitivity reactions remains limited and the complete immunological mechanisms of sensitization for QAC and other LMW sensitizers are not fully understood. However, much knowledge has been gained in the last decade regarding the mechanisms of related immune responses. Due to the complexity of mechanisms involved in chemical allergy and the lack of knowledge regarding these mechanisms, investigations of novel factors and molecules involved are imperative for the greater understanding of these conditions. While epidemiological studies support a role for QAC in allergic disease, these are the first animal studies to confirm that DDAB is a sensitizing chemical. In summary, based on calculated EC3 values from the LLNA, these studies demonstrate dermal sensitization potential (strong), based on the criteria set forth by ICCVAM (2011) for the disinfectant DDAB. While direct associations of dermal exposure to DDAB on human health have not been fully established, the above mentioned studies raise concerns about exposure to this chemical and the influence of exposure duration on allergic sensitization.

## Supplementary Material

1

## Figures and Tables

**Table 1.** Body/organ weights of female Balb/c mice dermally exposed for 14 days to DDAB.

DDAB (w/v)%				

Parameter	0	0.0625	0.125	0.25
Body weight (g)	21.91 ± 1.21	21.58 ± 0.58	21.99 ± 0.50	22.45 ± 1.12
Kidney weight (mg)	264 ± 2	264 ± 4	267 ± 5	286 ± 6
% bw^a^	1.20 ± 0.02	1.22 ± 0.04	1.21 ± 0.05	1.29 ± 0.05
Spleen weight (mg)	95 ± 5	93 ± 1	99 ± 5	91 ± 5
% bw	0.43 ± 0.02	0.43 ± 0.01	0.45 ± 0.02	0.41 ± 0.02
Thymus weight (mg)	37 ± 4	36 ± 3	33 ± 4	*^,#^24 ± 2
% bw	0.17 ± 0.01	0.17 ± 0.01	0.15 ± 0.02	**^,#^0.11 ± 0.005
Liver weight (mg)	1045 ± 66	992 ± 34	1041 ± 28	1114 ± 39
% bw	4.76 ± 0.11	4.60 ± 0.09	4.74 ± 0.09	4.98 ± 0.09

a bw: body weight.

Values are means (±SE) for each group. Value significantly different from acetone control at or

* *p* < 0.05

** *p* < 0.01.

Linear trend

# *p* < 0.05.

**Table 2.** Effects of dermal exposure to DDAB on DLN cell number and lymphocyte sub-populations in mice after four days exposure.

DDAB (%)				

Parameter	0	0.0625	0.125	0.25
DLN number (×10^7^)	0.28 ± 0.02	0.47 ± 0.04	1.04 ± 0.85**	1.29 ± 0.14**
B-cell number(×10^6^)	0.56 ± 0.07	1.02 ± 0.12	2.71 ± 0.26**	4.26 ± 0.62**
% of DLN	19.78 ± 1.45	21.68 ± 0.58	25.96 ± 0.73**	32.72 ± 1.35**
CD4^+^T-cell number(×10^6^)	1.52 ± 0.11	2.49 ± 0.25	5.16 ± 0.38**	5.65 ± 0.61**
% of DLN	54.24 ± 1.03	53.16 ± 0.57	49.78 ± 0.47**	44.12 ± 1.33**
CD8^+^T-cells(×10^6^)	0.46 ± 0.03	0.81 ± 0.09	1.55 ± 0.19**	1.85 ± 0.22**
% of DLN	16.54 ± 0.50	17.06 ± 0.40	14.76 ± 0.62	14.44 ± 0.47*
Dendritic Cells(×10^5^)	0.31 ± 0.01	0.42 ± 0.07	1.03 ± 0.04**	1.23 ± 0.13**
% of DLN	1.16 ± 0.11	0.89 ± 0.08	1.00 ± 0.04	0.96 ± 0.04

Mice were dermally exposed to vehicle (acetone) or different concentrations of DDAB for four consecutive days. The mice were euthanized six days after the final exposure, DLN were removed, and total cells counted. Numbers and frequency of B- and T-cells, and subsets of T-cells (CD4^+^ and CD8^+^), and dendritic cells were enumerated. Values represent the means (±SE) for each group. Significantly different from acetone controls at

* *p* < 0.05

** *p* < 0.01.

**Table 3.** Effects of dermal exposure to DDAB on DLN cell number and lymphocyte sub-populations in mice after 14 days exposure.

DDAB (%)				

Parameter	0	0.0625	0.125	0.25
DLN number (×10^6^)	0.348 ± 0.03	0.69 ± 0.07*	1.33 ± 0.10**	1.97 ± 0.09**
B-cell number(×10^6^)	0.79 ± 0.11	1.48 ± 0.17	3.70 ± 0.36**	6.70 ± 0.48**
% of DLN	22.56 ± 1.32	21.38 ± 0.70	27.70 ± 0.65**	33.86 ± 1.07**
CD4^+^T-cell number(×10^6^)	1.69 ± 0.14	3.52 ± 0.36**	6.14 ± 0.45**	8.00 ± 0.28**
% of DLN	48.98 ± 1.69	51.14 ± 0.92	46.30 ± 0.59	40.74 ± 0.86**
CD8^+^T-cells(×10^6^)	0.54 ± 0.06	1.15 ± 0.11**	2.14 ± 0.12**	3.24 ± 0.16**
% of DLN	15.64 ± 0.56	16.80 ± 0.51	16.22 ± 0.47	16.46 ± 0.33*
Dendritic Cells(×10^5^)	0.43 ± 0.03	0.78 ± 0.16	1.62 ± 0.18**	2.09 ± 0.19**
% of DLN	1.28 ± 0.11	1.10 ± 0.10	1.22 ± 0.08	1.08 ± 0.08

Mice were dermally exposed to vehicle (acetone) or different concentrations of DDAB for 14 consecutive days. The mice were euthanized six days after the final exposure, DLN were removed, and total cells counted. Numbers and frequency of B- and T-cells, and subsets of T-cells (CD4^+^ and CD8^+^), and dendritic cells were enumerated. Values represent the means (±SE) for each group. Significantly different from acetone controls at

* *p* < 0.05

** *p* < 0.01.

**Table 4.** Phenotypic and IgE analyses after *in vivo* DDAB treatments.

	B-cells (% lymphocyte population)		IgE^+^ B-cells (% total B-cells)		
Dose group	%	Cells (×10^6^)	%	Cells (×10^5^)	Total IgE (ng/ml)
4 days					
0%	19.78 ± 1.45	0.56 ± 0.07	5.74 ± 0.71	0.31 ±0.02	4255 ± 758
0.0625	21.68± 0.58	1.02 ± 0.01	8.30 ±2.00	0.80 ± 0.02	3268 ± 645
0.1250	25.96 ± 0.73**	2.71 ±0.26**	12.51 ±2.14	3.46 ±0.75	3815 ± 295
0.2500	32.72 ± 1.35**	4.26 ±1.38**	19.01 ±4.33**	8.91 ±2.93**	3585 ± 872
14 days					
0%	22.56 ± 1.32	0.79 ±0.11	8.85 ± 1.23	0.70 ±0.01	4479 ± 556
0.0625	21.38± 0.70	1.48 ±0.17	18.92 ±3.91	2.96 ± 0.85	4510 ±532
0.1250	27.70 ± 0.65**	3.69 ±0.36**	13.99 ±2.155	5.10 ± 0.77**	7226 ± 2238
0.2500	33.86 ±1.07**	6.69 ±0.48**	29.70±3.16**	20.10 ±2.92**	15 702 ± 4202**

Levels of significance denoted as compared to vehicle (acetone).

** *p* < 0.01

Values shown are means (*n* = 5)±SE.
