# Supplementary figures and images for: Divergent hypersensitivity responses following topical application of the quaternary ammonium compound, didecyldimethylammonium bromide
Source: J Immunotoxicol. Author manuscript; Available in PMC 2019 Feb 27. (PMC6391722; doi:10.1080/1547691X.2017.1397826)

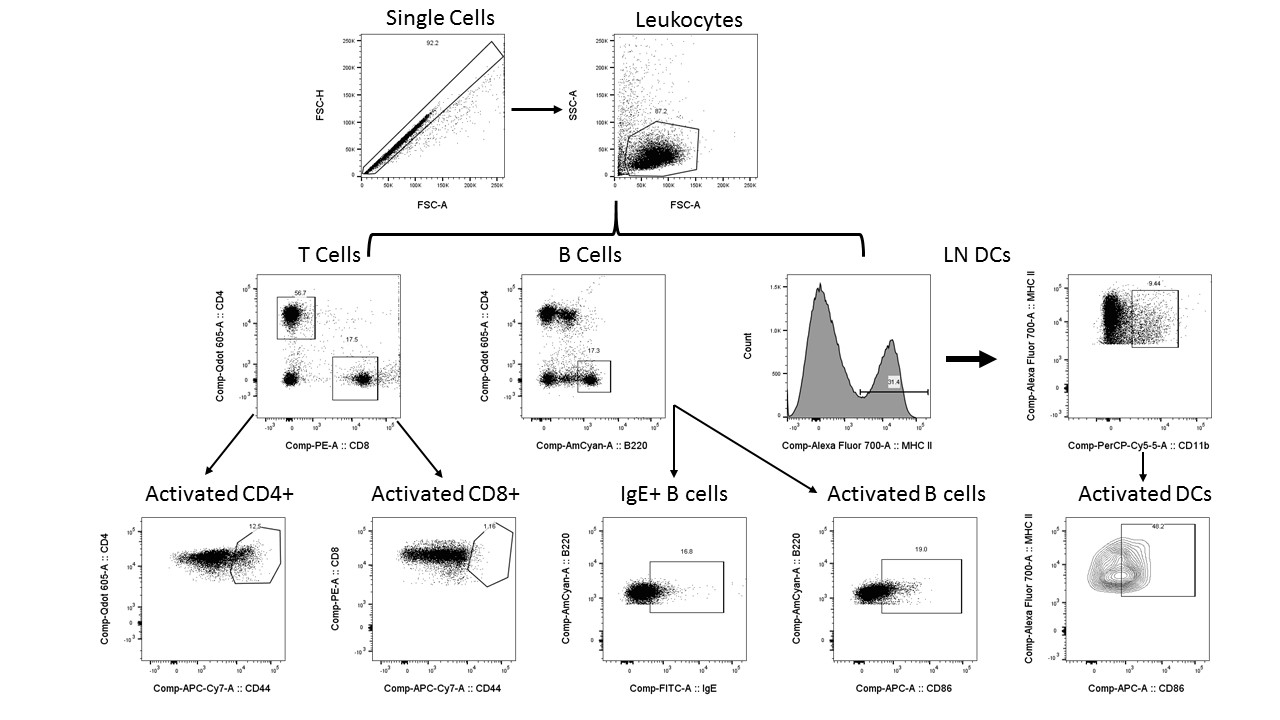

Supplement: 1 [file NIHMS1011403-supplement-1.jpg]
